# Genomic Loads and Genotypes of Respiratory Syncytial Virus: Viral Factors during Lower Respiratory Tract Infection in Chilean Hospitalized Infants

**DOI:** 10.3390/ijms18030654

**Published:** 2017-03-21

**Authors:** Yazmín Espinosa, Camila San Martín, Alejandro A. Torres, Mauricio J. Farfán, Juan P. Torres, Vasanthi Avadhanula, Pedro A. Piedra, Lorena I. Tapia

**Affiliations:** 1 Department of Pediatrics and Pediatric Surgery, Hospital Roberto del Río, Facultad de Medicina, Universidad de Chile, Profesor Zañartu 1085, Independencia, 8380418 Santiago, Chile; y.espinosa.v@gmail.com (Y.E.); camilasanmartin@gmail.com (C.S.M.); 2 Virology Program, Institute of Biomedical Sciences (ICBM), Facultad de Medicina, Universidad de Chile, Independencia 1027, Pabellón J. Independencia, 8380453 Santiago, Chile; a.torres.riquelme@gmail.com; 3 Department of Pediatrics and Molecular Biology Laboratory, Hospital Luis Calvo Mackenna, Facultad de Medicina, Universidad de Chile, Av. Antonio Varas 360, Providencia, 7500539 Santiago, Chile; mfarfan@med.uchile.cl (M.J.F.); jptorres@med.uchile.cl (J.P.T.); 4 Department of Molecular Virology and Microbiology, Baylor College of Medicine, One Baylor Plaza, Room 248E, Houston, TX 77030, USA; avadhanu@bcm.edu (V.A.); ppiedra@bcm.edu (P.A.P.); 5 Pediatrics, Baylor College of Medicine, One Baylor Plaza, Houston, TX 77030, USA

**Keywords:** respiratory syncytial virus, RSV genotypes, viral load, severity

## Abstract

The clinical impact of viral factors (types and viral loads) during respiratory syncytial virus (RSV) infection is still controversial, especially regarding newly described genotypes. In this study, infants with RSV bronchiolitis were recruited to describe the association of these viral factors with severity of infection. RSV antigenic types, genotypes, and viral loads were determined from hospitalized patients at Hospital Roberto del Río, Santiago, Chile. Cases were characterized by demographic and clinical information, including days of lower respiratory symptoms and severity. A total of 86 patients were included: 49 moderate and 37 severe cases. During 2013, RSV-A was dominant (86%). RSV-B predominated in 2014 (92%). Phylogenetic analyses revealed circulation of GA2, Buenos Aires (BA), and Ontario (ON) genotypes. No association was observed between severity of infection and RSV group (*p* = 0.69) or genotype (*p* = 0.87). After a clinical categorization of duration of illness, higher RSV genomic loads were detected in infants evaluated earlier in their disease (*p* < 0.001) and also in infants evaluated later, but coursing a more severe infection (*p* = 0.04). Although types and genotypes did not associate with severity in our children, higher RSV genomic loads and delayed viral clearance in severe patients define a group that might benefit from new antiviral therapies.

## 1. Introduction

Respiratory syncytial virus (RSV) is the most common cause of lower respiratory tract infection (LRTI) in children [1] and an important agent in the elderly and immunocompromised patients [2], generating a global public health problem [3]. In Chile, RSV is the cause of important outbreaks each winter, accounting for a high number of outpatient visits and hospitalizations [4]. Actual treatment is mainly supportive [5,6] and although an immunoprophylactic therapy (palivizumab, Synagis^®^, MedImmune, Inc., Gaithersburg, MD, USA) has been approved for infants at high risk of severe infection [7], most hospitalized RSV infected infants do not meet the criteria for immunoprophylaxis. Moreover, a high-risk condition is not present in approximately 65% of RSV hospitalized infants [1], making it difficult to predict who will develop a severe disease. The pathogenesis of RSV remains the topic of intense basic and clinical research with emphasis on the host (genetic and immunologic), viral, and environmental factors in relation to severity of infection.

RSV has been classified into groups A and B based on antigenic differences of the G, F, and N proteins [8]. Further genetic analysis of the nucleotide sequence of the second hypervariable region on the G gene allowed for the classification into genotypes within antigenic groups RSV-A and B [9,10]. Many studies have reported the molecular epidemiology and genetic variability of RSV worldwide [10,11,12,13,14,15,16,17,18,19,20], revealing the emergence and spread of new genotypes with duplication of ~60 to 70 nucleotides in this G hypervariable region for RSV-A (RSV Ontario) and RSV-B (RSV Buenos Aires) [15,19,21,22]. Although none of the variants or genotypes has been consistently associated with greater virulence or pathogenicity [13,18,22,23], some recent reports describing clinical aspects of contemporary genotypes (NA1 and ON1) have shown higher frequencies of LRTI and hospitalizations rates [24,25,26].

Another viral factor that has been explored as determinant of respiratory disease severity is the viral load in the respiratory tract. Some studies have detected a correlation between RSV loads and the disease severity in hospitalized infants [27,28], and others have reported that higher viral loads predict longer durations of hospitalizations [29,30]. The latest was recently confirmed by a multicenter prospective cohort including 1764 children with RSV bronchiolitis [31]. Thus, early intervention with antiviral therapies initiated at the time of highest viral replication (approximately at the third to fifth day of symptoms) could improve the prognosis of the disease, directly reducing the viral cytopathic effects and inflammatory response [30].

The aim of this study was to describe molecular viral factors: RSV group, genotype, and viral genomic loads in Chilean infants during two consecutive RSV epidemics (2013 and 2014) and explore their relationship with severity of disease.

## 2. Results

### 2.1. Patients and Clinical Severity

A total of 90 hospitalized infants with RSV LRTI were recruited, 40 cases during the 2013 outbreak and 50 during the 2014 outbreak in Santiago. We excluded four immunofluorescence assay IFA positive RSV cases because they could not be confirmed by Q-PCR. Thus, data from 86 infants were available for the analysis. The median age was 73.7 (14–247) days and 54.6% were male. Based on the clinical score, 49 infants were determined to have moderate and 37 severe disease, with similar frequencies between the 2013 and 2014 epidemic outbreaks (*p* = 0.63). In Table 1, demographics, medical history, and clinical characteristics by severity of the infection are presented. No significant differences were found in age, gender, or medical history. Length of stay, requirement of supplemental oxygen, FiO_2_ > 30%, and mechanical ventilation were significantly higher in the severe group.

### 2.2. Viral Diagnosis

RSV was the only pathogen detected in 66 cases (76.7%), and coinfection was observed in 20 patients (23.3%). The coinfection patterns were: 16 cases with RSV-human rhinovirus (HRV) (80%); 2 cases with RSV-coronavirus (CoV) HKU1 (10%), 1 case with RSV-parainfluenza virus type 3 (PIV3) (5%), and 1 case with RSV-HRV-bocavirus (5%). No significant associations were found between demographics or clinical features and coinfection (Table 2). Nevertheless, it was observed that coinfection was more frequent in patients with a family history of asthma or atopy (*p* = 0.017).

### 2.3. RSV Groups and Genotypes

**Figure 1:**
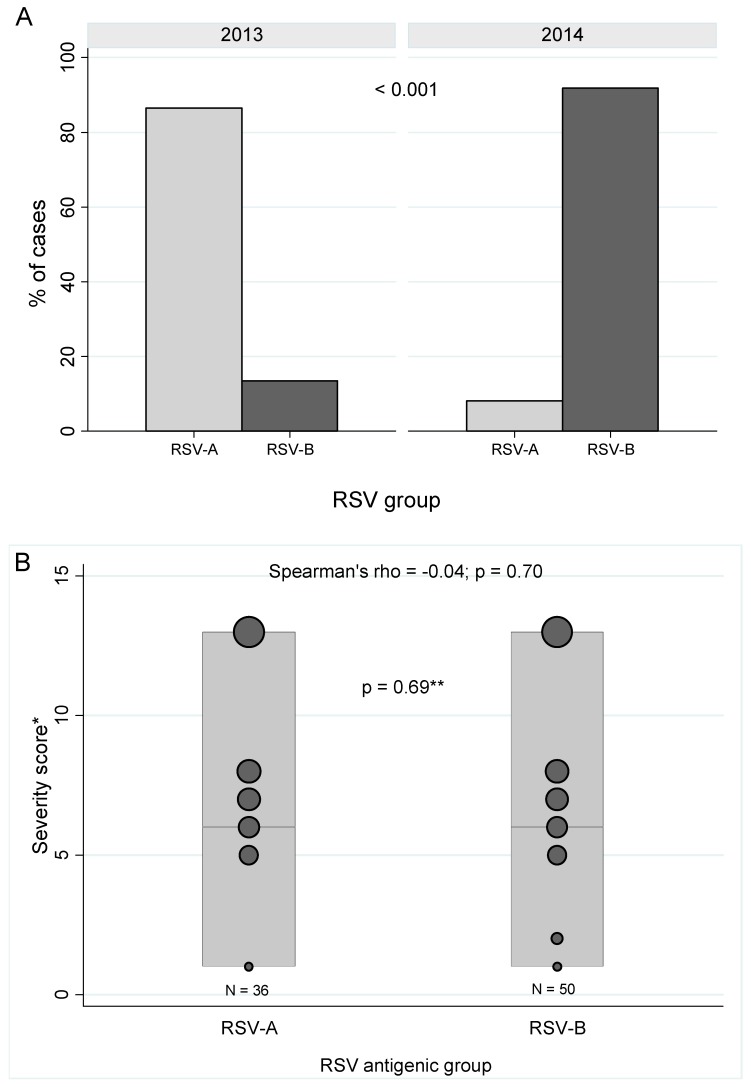

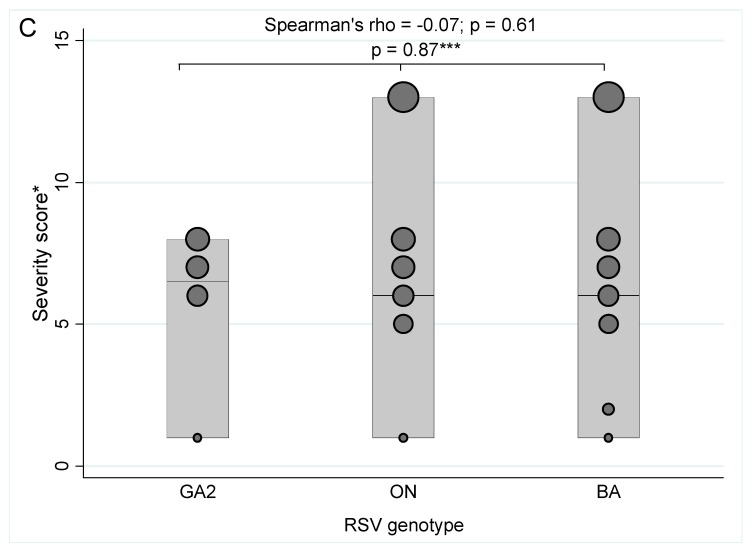
RSV antigenic groups and genotypes detected in hospitalized infants with LRTI. (**A**) RSV antigenic groups detected during 2013 and 2014 epidemic outbreaks in Santiago, Chile; (**B**) Severity of the infection by antigenic groups: Median, Minimum, and Maximum values are represented in bars. Scatter plot with weighted markers is shown with circles; (**C**) Severity of infection by genotypes (ON: Ontario; BA: Buenos Aires): Median, Minimum, and Maximum values are represented in bars. Scatter plot with weighted markers is shown with circles. * Severity score by Larrañaga et al., 2009 [32]. ** Mann-Whitney test. *** Kruskal-Wallis test.

Antigenic groups RSV-A (*n* = 36) and RSV-B (*n* = 50) were detected, showing different circulation frequencies when comparing the two epidemic seasons (*p* < 0.01). As shown in Figure 1A, during the 2013 outbreak, RSV-A strains were dominant with a frequency of 86% (*n* = 32). In contrast, during 2014 the major group was RSV-B, found in 92% (*n* = 45) of patients. No difference in the severity of the infection was detected between antigenic groups (Figure 1B, *p* = 0.69). Moreover, no significant correlation was found between RSV groups and severity (Spearman’s *rho* = −0.04; *p* = 0.70).

Genotype determination was performed on 55 samples (64%). Sequenced viruses in RSV-A clustered with reference viruses in genotypes GA2 (*n* = 6) and ON (*n* = 17) (Supplementary Figure S1). All RSV-B viruses clustered with reference viruses in genotype BA (*n* = 32), as shown in Supplementary Figure S2. We found no difference in disease severity between GA2, ON, and BA genotype (Figure 1C, *p* = 0.87) with no significant correlation (Spearman’s *rho* = −0.07; *p* = 0.61).

### 2.4. Respiratory Syncytial Virus (RSV) Genomic Loads

**Figure 2:**
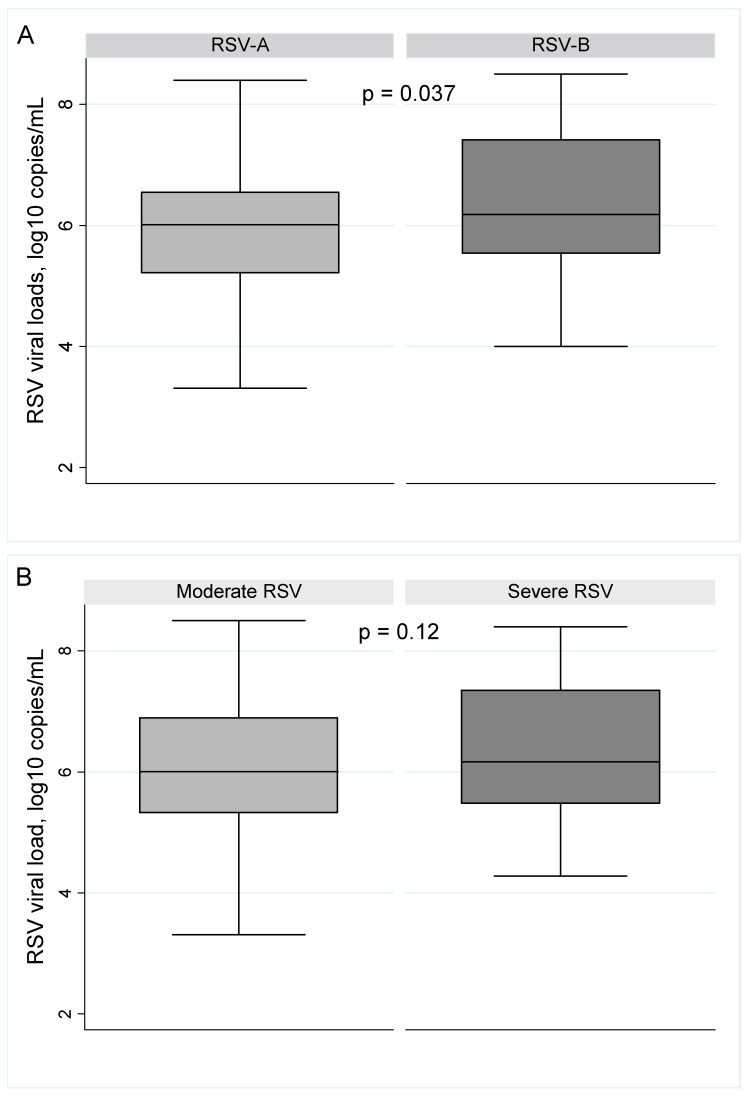
RSV genomic loads detected in hospitalized infants with LRTI. (**A**) Viral loads by RSV antigenic groups; (**B**) Viral loads by the severity of the infection.

A significant difference in viral genomic load was not detected between RSV infected infants having RSV as the sole infection or as a coinfection (6.18 ± 1.18 vs. 6.12 ± 1.01 log10copies/mL; *p* = 0.41). Interestingly, a significant difference in the respiratory viral genomic load was detected between the RSV antigenic groups (Figure 2A; *p* = 0.037). RSV-B infected infants had a higher median viral genomic load (6.36 ± 1.14 log10copies/mL) than RSV-A (5.91 ± 1.10 log10copies/mL) infected infants. Although higher viral genomic loads were detected in nasal secretions of severe cases (6.34 ± 1.19 vs. 6.04 ± 1.09 log10copies/mL), the difference was not statistically significant (Figure 2B, *p* = 0.12).

**Figure 3:**
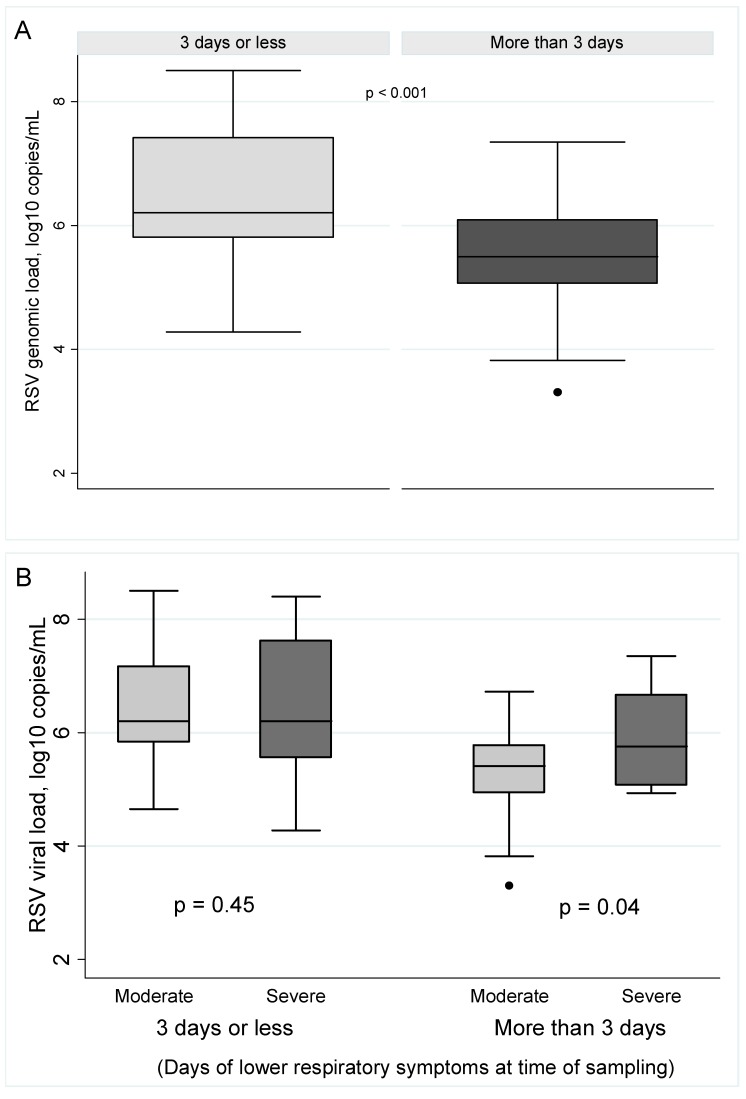
RSV genomic loads detected in hospitalized infants with LRTI by duration of lower respiratory symptoms at the time of sampling. (**A**) Viral loads in patients with early (3 days or less of lower symptoms) and late (more than 3 days of symptoms) RSV infection; (**B**) Viral loads in patients with early and late illness according to the severity of RSV infection.

Viral genomic loads were also analyzed by the duration of the LRTI at the time of sampling. As illustrated in Figure 3A, significantly higher viral genomic loads were found in infants with 3 days or less of lower respiratory tract symptoms compared to those with more than 3 days (*p* < 0.001). Thus, higher viral loads were detected in the RSV infected infants with an earlier LRTI presentation (6.44 ± 1.09 vs. 5.52 ± 0.98 log10copies/mL). Finally, analyzing the stage (≤3days, >3 days) of LRTI presentation by severity of infection (Figure 3B), we found that viral genomic loads at day 3 or less of LRTI were comparable between moderate and severe LRTI (6.42 ± 0.97 vs. 6.45 ± 1.24 log10copies/mL; *p* = 0.45), however, in the later LRTI stage (≥3 days) the infants with severe RSV LRTI had significantly higher viral genomic loads (5.26 ± 0.92 vs. 5.98 ± 0.95 log10copies/mL; *p* = 0.04).

Independent viral factors of RSV disease severity were explored by bivariate (Table 3) and multivariate analyses. RSV antigenic group, genotype, or viral genomic load did not predict severity of infection. A higher odds ratio, although not statistically significant, was detected when the RSV genomic load was analyzed with respect to the duration and severity of the LRTI with a higher viral genomic load in infants with longer duration of severe LRTI (OR = 2.51, *p* = 0.09). This association did not change when adjusted by age, gender, and RSV type (data not shown).

## 3. Discussion

Recent promising reports on the development of drug therapeutics for RSV have been published [33,34] in response to a critical medical need for the care of infants with moderate to severe RSV illness. In parallel, molecular virology has enhanced our ability to dissect the viral contribution to the severity of the disease. However, many questions remain unclear about the direct role of RSV in airway injury and clinical outcome. In this study, we present evidence about viral factors—RSV group, genotype, and viral genomic load—that might affect the course and severity of the disease.

Circulation of RSV in Santiago de Chile has been reported by Avendaño et al. [4], who described cocirculation of RSV-A and RSV-B antigenic groups with dominance of A in alternate years. They also described that epidemics appeared earlier and of greater intensity when RSV-B dominated. Our results in two consecutive years showed this alternating antigenic pattern, with RSV-A being dominant during 2013 and RSV-B in 2014. Moreover, as reported by the Institute of Public Health (ISP) in Chile [35], an earlier RSV epidemic occurred in 2014, when viruses in the RSV-B group were the major circulating RSV.

No association of the antigenic group (A or B) with severity of the disease was observed in our study, which is in agreement with the majority of the publications in this area.

Regarding RSV genotypes, as expected and according to the global transmission dynamics described by Duvvuri et al. [19], the recently described RSV-A Ontario genotype [15] was detected in our population since 2013, replacing all RSV-A samples collected in 2014. For RSV-B, only BA genotype was identified. In our hospitalized patients, no association was found between the circulating genotypes (GA2, ON, BA) with the severity of the lower respiratory tract disease. In contrast to recent reports [24,25,26], the recently described ON genotype did not show a higher severity score. As with the influenza virus, continuous surveillance may be needed to detect new emergent genotypes and to assess their immediate effect in susceptible populations. We have recently described that genetic variations are present in the three RSV surface proteins [16], including not only G protein, but also the fusion (F) protein, which is the major determinant for the host’s immune response, and the main target for antiviral therapy and vaccination strategies [36]. Some of these genomic variations in the viral genome might impact the pathogenic process and the disease, and thus need to be explored beyond genotype classification.

In our study, a relationship between coinfection with other viruses and severity was not detected when compared to infants with sole RSV infection. Moreover, there was a trend, although not significant, for less severe RSV disease with the presence of coinfection with other viral agents (*p* = 0.063). This finding may be explained, in part, by the older age of the coinfected group (74.5 vs. 52 days; *p* = 0.08), but it is also relevant that 80% of these patients had RSV-HRV infection. It has been shown that infants with HRV bronchiolitis are older, and more likely to have asthma-like characteristics [37,38,39], which is also in agreement with the association detected with family history of asthma or atopy in our group with RSV coinfection. 

Recent publications have described associations between RSV loads and severity scores [40,41], duration of hospitalization [30,31], and Intensive Care Unit requirements [30]. Moreover, analyzes considering the days of symptoms prior to sample collection have shown that viral clearance is an important factor to consider [30,41], since significant differences are detected in genomic loads and their predictive capacity of severity depending on the date of sampling. In our study, we confirmed that a greater number of RSV genomic copies are found when sampling occurs early during the LRTI, being significantly lower when the samples are collected later than 3 days of onset of cough, the symptom we chose as a marker of LRTI. In agreement with the studies by Zhou et al. [41] and El Saleeby et al. [30], we found higher viral loads in the infants with severe LRTI with more than 3 days of lower respiratory tract symptoms, but not in infants with severe LRTI at 3 days or less of illness, suggesting a difference in the kinetics of viral clearance. We postulate that a delayed viral clearance is contributing to greater airway injury and longer duration of severe LRTI. Although the main limitation in this study is the lack of sequential samples from our patients, we believe that the data reported complements the limited information collected by other authors. We propose a simple clinical approach with cough as a marker to characterize a population that may benefit from possible future therapeutic approaches. The data presented in relation to genotypes and viral loads should drive further exploration of viral evolution and viral dynamics during primary RSV infection and its relation to severity.

## 4. Materials and Methods

An Observational, Analytical, Cross-Sectional Study Was Performed.

### 4.1. Patients

By consecutive sampling, infants under 1 year of age who presented with their first community-acquired RSV LRTI were enrolled after obtaining parental (legal guardian) written informed consent. They were hospitalized at Hospital Roberto del Río in Santiago de Chile, during winter outbreaks (May to September) of 2013 and 2014. Inclusion criteria were: first documented episode of acute LRTI, a positive direct immunofluorescence assay for RSV (performed at HRR central laboratory at admission), and enrollment into the study within the first 72 h of hospital admission. Infants with possible bacterial infection, prematurity (<37 weeks gestational age), immunodeficiency, and documented chronic lung or heart diseases were excluded.

### 4.2. Samples

A single nasopharyngeal aspirate (NPA) sample was obtained according to the standard of respiratory care. The samples were refrigerated and transported to the Virology Laboratory during the day of collection and were divided into aliquots in order to perform viral diagnostic confirmation, genotype analysis, and viral genomic load quantification.

### 4.3. Demographic and Clinical Characteristics

Demographic and clinical data were collected by a structured interview with the parents or legal guardians at enrollment. The date of onset of lower respiratory symptoms (defined by the day the cough started) and the duration of lower respiratory disease at the time of sampling were also registered. In addition, after discharge from the hospital, data from the medical records were extracted and used to determine the severity of the illness, moderate or severe, based on a previously published clinical score [32].

### 4.4. Viral Diagnosis and Determination of RSV Genomic Load

Singleplex or duplex 2-step real time PCR assays and analyses were performed at the laboratories of L.I.T and P.A.P using primers and probes that have been previously described [42,43,44]. Real time reverse transcription-PCR (Q-PCR) was used to detect RNA viruses, including: RSV-A and RSV-B; human rhinovirus (HRV), coronaviruses (CoV) NL-63, HKU1, OC43, and 229E; parainfluenza virus (PIV) types 1, 2, and 3; influenza virus types A, B, and 2009 A (H1N1); human metapneumovirus (HMPV). Real time-PCR was performed for the detection of DNA pathogens including adenovirus, bocavirus, Mycoplasma pneumoniae, and Bordetella pertussis. Viral genomic loads for RSV positive samples were determined at the laboratory of P.A.P by Q-PCR. Threshold cycle (CT) values were determined, thereby providing a semiquantitative measure of viral genomic load, as previously described [45].

### 4.5. RSV Genotype Determination

Amplification of the second hypervariable region of the G gene was performed using primers and conditions of PCR reactions as previously described [16]. PCR products were purified and sent for sequencing by Sanger method at Macrogen Inc. (Seoul, Korea). Chromatogram traces were visualized with BioEdit Sequence Alignment Editor Version 7.0.9.0 [46] to confirm the quality score. For contig assembling, SeqMan^®^ program of Lasergene 8 program suite (DNASTAR, Inc., Madison, WI, USA) was used, obtaining the G gene sequence for each strain. A fragment of ~270 to 330 bp located in the second hypervariable region of the G gene was obtained. Based on the methodology described by Peret et al. [9], phylogenetic analyses were performed with R software (R Development Core Team, 2009) including our sequences and reference sequences retrieved from Genbank [9,10,11,12,14,47,48,49]. RSV group and genotype categorization was achieved based on the clustering and distribution within the phylogenetic tree [9,16].

### 4.6. Statistical Analysis

Demographic and clinical characteristics were described using frequencies in the case of categorical variables, and measures of central tendency and dispersion for continuous variables. Comparisons between groups were performed using χ^2^, Mann-Whitney Rank Sum, or Kruskal Wallis tests as appropriate. A *p*-value of <0.05 was considered statistically significant. Spearman correlation test was used to analyze relationship between viral types and severity. The association between clinical severity and independent variables was assessed by χ^2^ tests and logistic regression analysis.

### 4.7. Ethics

This FONCECYT study N°11121536 was approved by the Local Ethics Committee of the North Metropolitan Health Service (25 October 2012) and the Faculty of Medicine, Universidad de Chile (28 June 2012). Informed consent was signed by the parents of all study participants.

## 5. Conclusions

Although neither the RSV antigenic group nor the genotypes were associated with clinical severity, a delayed viral clearance might be associated with greater airway injury and thus disease severity. New antiviral drug therapies hold the promise of shortening the clearance of RSV in the respiratory tract and thus might mitigate severity of disease.

## Figures and Tables

**Table 1:** Demographic characteristics, medical history, and clinical features of infants with moderate and severe RSV infection.

Patients Characteristics	Moderate RSV Infection (*n* = 49)	Severe RSV Infection (*n* = 37)	*p*-Value
Gender (% male)	46.9	64.8	0.1 ^a^
Age (days) ^b^	52 (14–242)	55 (15–247)	0.5 ^c^
**Medical history**			
Breastfeeding (%) ^d^	91.8	91.9	0.99 ^a^
Personal atopic history (%)	6.1	2.7	0.46 ^a^
Family history of asthma or atopy (%)	18.4	24.3	0.5 ^a^
Maternal smoking (%)	22.5	32.4	0.3 ^a^
Indoor smoke (%) ^e^	48.9	64.9	0.14 ^a^
Siblings (n°) ^b^	1.35 (0–6)	1.03 (0–5)	0.13 ^c^
**Clinical features**			
Length of stay (days) ^b^	4.04 (2–11)	9.62 (4–36)	<0.001 ^c^
Supplemental oxygen requirement (%)	85.7	100	0.02 ^a^
FiO_2_ > 30% (%)	6.1	45.9	<0.001 ^a^
Mechanical ventilation (%)	0	10.8	0.02 ^a^

RSV, respiratory syncytial virus; ^a^ Χ^2^ test; ^b^ Median (Min-Max); ^c^ Mann-Whitney Rank Sum Test; ^d^ Breastfeeding at the moment of hospitalization; ^e^ Indoor smoking or combustion heating (wood, kerosene).

**Table 2:** Clinical characteristics and medical history of infants with RSV lower respiratory tract infection (LRTI) as a single infection and coinfection.

Patients Characteristics	RSV Single Infection (*n* = 66)	RSV + Other Virus (*n* = 20)	*p*-Value
**Clinical data:**			
Age (days) ^a^	52 (14–247)	74.5 (17–242)	0.08 ^b^
Severe infection (%)	48.5	25	0.063 ^c^
Length of hospitalization (days) ^a^	6 (2–36)	4 (2–17)	0.2 ^b^
**Medical history:**			
Personal atopic history (%)	3.03	10	0.195 ^c^
Maternal smoking (%)	25.8	30.0	0.71 ^c^
Indoor smoke (%) ^d^	57.6	50	0.55 ^c^
Familiar asthma or atopic history (%)	15.1	40	0.017 ^c^

RSV, respiratory syncytial virus; ^a^ Median (Min-Max); ^b^ Mann-Whitney rank sum test; ^c^ Χ^2^ test; ^d^ Indoor smoking or combustion heating (wood, kerosene).

**Table 3:** Demographics, viral types, and viral loads as independent factors predicting severity in infants with RSV infection.

Severe RSV Infection		
Independent factors	OR (95% CI)	*p*-Value
Male gender	2.09 (0.87–5.02)	0.1
Age (days)	1.00 (0.99–1.00)	0.59
RSV antigenic type	1.10 (0.46–2.62)	0.82
RSV genotype ^a^		
GA2	1.07 (0.26–4.28)	0.93
ON	1.07 (0.38–3.06)	0.89
BA	1.19 (0.50–2.80)	0.68
RSV genomic loads		
All cases (*n* = 86)	1.26 (0.86–1.86)	0.23
Early disease ^b^ (*n* = 61)	1.02 (0.65–1.63)	0.90
Late disease ^c^ (*n* = 25)	2.51 (0.85–7.41)	0.09

RSV, respiratory syncytial virus; OR: odds ratio; CI: confidence interval; ON: Ontario; BA: Buenos Aires; ^a^ Reference value = “other genotypes”; ^b^ Early disease: 3 days or less of lower respiratory symptoms; ^c^ Late disease: more than 3 days of lower respiratory symptoms.

## Supplementary Materials

Supplementary materials can be found at www.mdpi.com/1422-0067/18/3/654/s1.
