# Supplementary material for: Genomic Loads and Genotypes of Respiratory Syncytial Virus: Viral Factors during Lower Respiratory Tract Infection in Chilean Hospitalized Infants
Source: Int J Mol Sci. 2017 Mar 21;18(3):654. doi: 10.3390/ijms18030654 (PMC5372666; doi:10.3390/ijms18030654)
Supplement: Supplementary file 1 [file ijms-18-00654-s001.pdf]

Supplementary Material

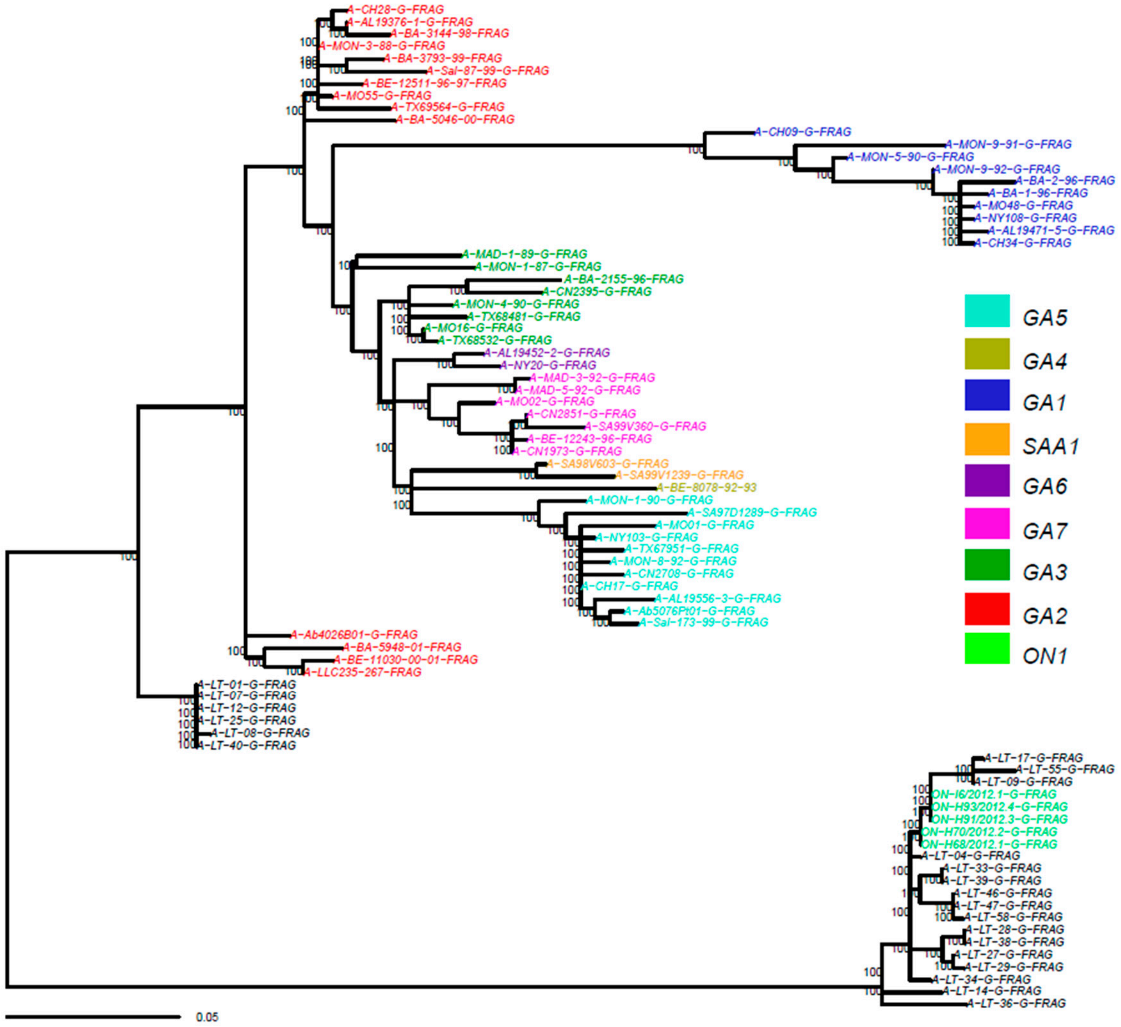

Figure S1. RSV Phylogenetic trees of RSV strains group A, based on the second hypervariable region of G-gene as described by Peret et al [9]. Selected worldwide previously described sequences (color), were retrieved from GenBank and compared to local strains from Santiago, Chile (black). Phylogenetic tree construction was performed by maximum likelihood analysis, and bootstrap values were calculated to support clustering. Only bootstrap values greater than 75% are shown.

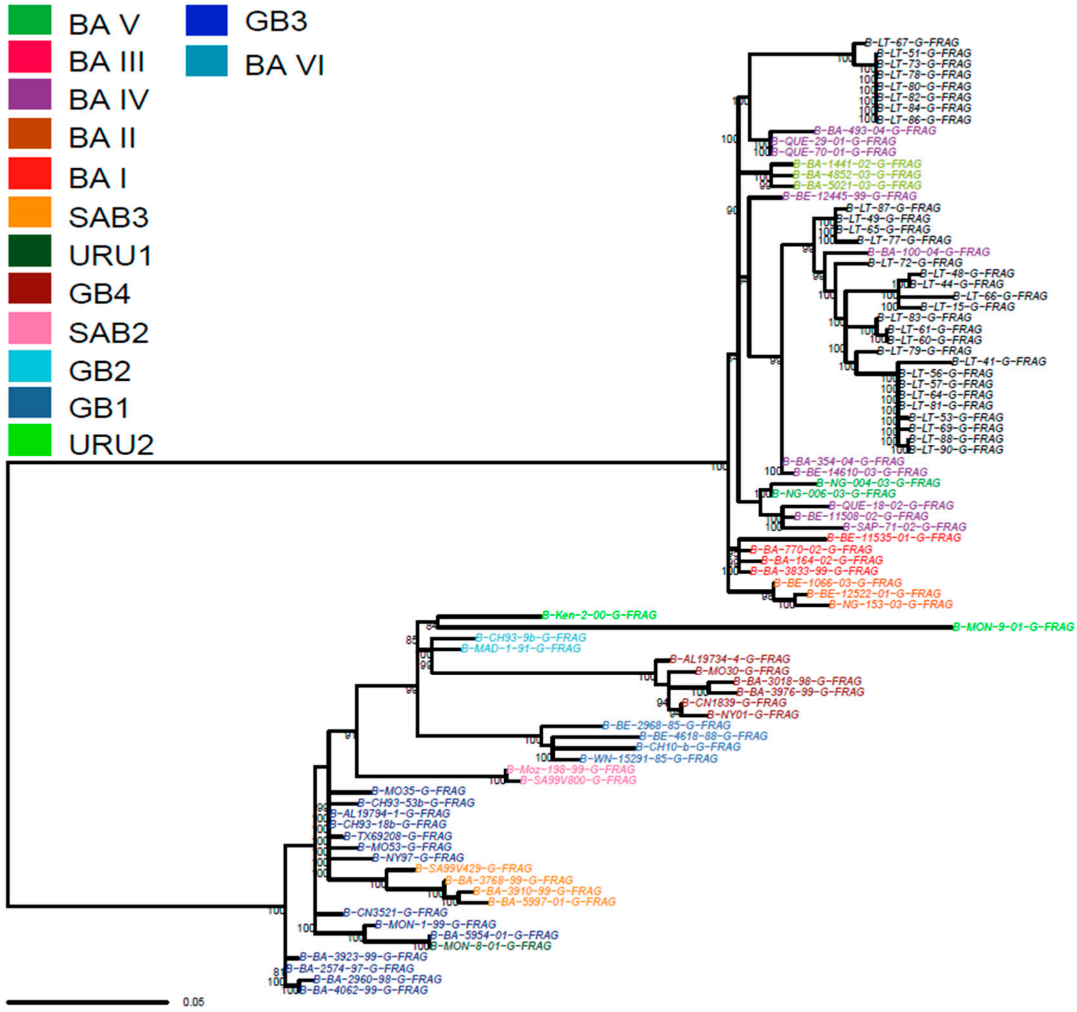

Figure S2. RSV Phylogenetic trees of RSV strains group B, based on the second hypervariable region of G-gene as described by Peret et al [9]. Selected worldwide previously described sequences (color), were retrieved from GenBank and compared to local strains from Santiago, Chile (black). Phylogenetic tree construction was performed by maximum likelihood analysis, and bootstrap values were calculated to support clustering. Only bootstrap values greater than 75% are shown.
